# Care for caregivers- a mission for primary care

**DOI:** 10.1186/s12875-021-01579-6

**Published:** 2021-11-16

**Authors:** Aya Biderman, Sara Carmel, Shimon Amar, Yaacov G. Bachner

**Affiliations:** 1 Department of Family Medicine and Siaal Research Center for Family Medicine and Primary Care, Faculty of Health Sciences, Ben-Gurion University of the Negev, Ben-Gurion University of the Negev, POB 653, 84105 Beer-Sheva, Israel; 2 Clalit Health Services, Southern district, Beer-Sheva, Israel; 3 Department of public health, Faculty of Health Sciences, Ben-Gurion University of the Negev, Beer-Sheva, Israel; 4 Center for Multidisciplinary Research in Aging, Faculty of Health Sciences, Ben-Gurion University of the Negev, Beer-Sheva, Israel

**Keywords:** Caregivers, Elderly, Physicians, Preventive medicine, Primary health care

## Abstract

**Background:**

The number of elderly people living in the community who are limited in daily activities is increasing worldwide. This generates prolonged care, which usually falls on one family member, the family caregiver. Caregivers are prone to develop psychosocial and physical symptoms. As a result, the World Health Organization (WHO) issued a clear directive to assess and support these caregivers.

The main goals of this study were to assess primary care physicians’ (PCP) awareness to caregivers’ health risks and the extent that they recommended preventive measures to maintain the health of the caregivers. As no suitable instrument existed, a secondary goal was to develop a scale to measure physicians’ awareness to caregivers’ health and preventive treatment and test it’s psychometric properties.

**Methods:**

Data were collected from a convenience sample of 201 PCP interviewed with structured questionnaires.

**Results:**

The participants’ mean age was 48.5 ± 11.2 years and 53.5% were female. Only 48.5% were Israel medical graduates and 72% were board-certified family physicians. Nearly 34% had been primary caregivers of family members.

Most physicians (83.6%) were aware of the primary caregiver’s high-risk for morbidity and mortality, and recommended preventive care. On a multivariate regression, PCP's higher level of risk awareness, their country of medical school and board certification were significant for explaining recommendations for preventive care. However, being a primary caregiver for a sick family member neither contributed significantly to the physicians’ awareness to caregiving risks nor to their preventive care.

**Conclusion:**

Although a high percentage of physicians were aware and concerned about caregivers’ health, their preventive care activities were relatively passive. PCPs should take a more active and preventive role for maintaining caregivers’ health.

**Supplementary Information:**

The online version contains supplementary material available at 10.1186/s12875-021-01579-6.

## Background

The responsibility for prolonged care for elderly sick patients usually falls on family members known as “informal caregivers”. This care is usually provided by a family member who takes charge of treatment and devotes most weekly hours to patient care [1]. This individual is known as the primary caregiver. Caregiving ranges from providing direct care to the care recipient to complex health care and managing social service systems. Most primary caregivers are women [2, 3]. Some of them, in particular elderly spouses, are themselves sick or have significant physical or mental disabilities. Others, such as the children of the patient, are at critical stages of their own personal or professional lives and lack the time, the tools, or the skills needed to care for their sick relatives [2, 4].

Caregivers often suffer from psychosocial symptoms such as anxiety and depression [3, 5–7] which occur in up to 52% and is particularly common among the patients’ spouses [5]. In fact, depression is the most frequent negative health outcome among family caregivers [8]. The severity of depression among caregivers is often greater than among the sick patients themselves [9]. Compared to non-caregivers, in various age groups, physical symptoms are more commonly described, such as fatigue, digestion problems [10, 11] reduced immune system activity, slower wound healing [12], relatively higher blood pressure levels, and multiple sleep problems [13]. The task of caregiving also affects the caregivers’ social life, 50% of whom report a decrease in social ties due to the caregiving demands [14]. These findings are exacerbated when the caregivers are elderly themselves [15, 16], hence defined as a group at high risk, even termed in the literature “the hidden victims” [17]. Therefore, the World Health Organization (WHO) issued a clear directive calling to support and look after their welfare during the caregiving period and following the patient’s death [18].

The increase in life expectancy is particularly significant in western countries with low birth rates. Limitations in activities of daily living are frequent among 65+ aged group, and yet increasing [19]. Latest statistics in Israel showed that 11.8% of the general population were aged 65 years or older, that they live in one of four households in the country, and that 26% of whom are limited in daily activities (21.4% men and 30.2% women) [20]. These figures have a significant impact on health, economy and welfare systems on both national and individual levels.

The present Covid 19 pandemic has drastically affected the world health status and has altered the lives of millions of people, especially the older population [21]. Unfortunately, the death toll among the sick elderly is the greatest, and many more old people face social isolation, loneliness, depression and fear. In such circumstances, family members are expected to take the role and responsibility as caregivers. A recent study from Italy, found that quarantine induces a rapid increase of behavioral and psychological symptoms in approximately 60% of dementia patients and stress-related symptoms in two-thirds of their caregivers [22].

These principles direct family physicians to identify the caregivers of their patients, assess their health condition regularly, support, treat and refer them to appropriate resources for help [23, 24]. A recent review conducted on 27 papers published between the years 2009–2019, [25] described family physicians’ perspectives regarding their role in supporting family caregivers. The authors concluded that primary care is the ideal context for reaching most caregivers and that caregivers would benefit from their support. Yet, this review is limited by the paucity of information on primary care support for caregivers from the perspective of primary care physicians. Many of the reports included perspectives of a limited number of physicians. Better appreciation of primary care physicians and primary team members is required to provide actual data about their attitudes, beliefs, level of knowledge, perceived barriers and perceptions of the support that caregivers require [26, 27]. This review concluded that physicians’ perspectives about caregiver interactions are needed to enable health and community service planning by policy makers.

The main goals of this study were to assess primary care physicians’ awareness to family caregivers’ health risks due to caregiving and the extent that they take preventive measures to maintain caregivers’ health. Additionally, we identified physicians’ characteristics associations with awareness and preventive measures. As no suitable instrument existed, a secondary goal was to develop a scale to measure physicians’ awareness to caregivers’ health and preventive treatment and test it’s psychometric properties.

## Methods

***Study type:*** A cross sectional study.

### Study population and procedure

Data were collected from a sample of primary care physicians (PCPs) who were defined as board certified physicians or trainees in family or internal medicine, or general practitioners with at least 6 months experience who worked in a variety of clinics in Israel. Prospective participants were contacted either through the Association of Family Doctors lists or by direct contact. They were notified about the study purposes, that participation was voluntary, and that the data will be used for research purposes only. Those who agreed underwent a 40-min interview conducted by experienced interviewers. Data collection occurred prior to the Covid-19 pandemic. The study was approved by the Ethics Committee of Ben-Gurion University of the Negev (approval # 21–2014). It was exempted by the Ethics committee from signing informed consent forms.

### Measures

The study instrument:

As no suitable instrument for measuring physicians’ awareness to caregivers’ health and preventive treatment existed in the literature, we developed dedicated questionnaire for this study. For this purpose, we first conducted an in-depth literature review on the subject. Based on this review, three domains were identified: awareness of the risks of caregiving for the caregiver; recommendations by physicians on preventive care for the caregiver; and monitoring of the caregiver by the physician. Following these findings, we generated an initial number of 20 items pertained to those three domains. These items were reviewed by four healthcare professionals (two physicians and two medical sociologists) experts in the field of informal caregiving, to ascertain face validity. We asked the professionals to rate each item on a Likert scale of 1–10 based on the items’ relevance for each domain. Based on their scoring and comments, items with an average score lower than 7 were removed from the questionnaire. Finally, the first domain (awareness of the risks of caregiving for the caregiver) included 7 items, the second domain (recommendations by physicians on preventive treatment for the caregiver) 4 items, and the third domain (monitoring of the caregiver by the physician) 3 items (see Supplementary file 1). For each item of the three domains, the physicians were asked to rate the degree to which they agreed, on a Likert scale ranging from 1 (no) to 6 (always) for awareness and recommendations domains or 1 (never) to 3 (often) for the monitoring domain. For ‘awareness’ and ‘recommendations’, the mean of the different items was calculated as a general index of the extent of the element tested, and the sum of the items for ‘monitoring’.

Socio-demographic characteristics of the physicians included age (years), gender (male/female), family status (married/ cohabiting/ single/divorced), number of children, country of birth (Israel/other), and religiosity (secular/traditional/religious/ultra-religious).

Own caregiving experience of the physicians included: the existence of a dependent elderly family member/s (yes/no), being a primary caregiver for severely ill elderly family member/s (yes/no) and duration of caregiving (months).

Professional characteristics included experience as a physician (years), experience as a primary care physician (years), number of working hours (weekly), country of medical school graduation (Israel/other), board certified physician (yes/no), area of specialization (family medicine/other), and current place of work (HMO clinic, independent clinic, other).

### Statistical analysis methods

Descriptive statistics (mean, median, standard deviation, range) was used to describe the study measures. Associations between dependent and independent variables were tested by Pearson, Chi-square, or Spearman as appropriate for the type of variable. Only variables that correlated significantly with the physicians’ awareness of the risks of caregiving in the univariate analyses were included as independent variables in the multivariate analyses. Physicians’ recommendations on preventive care for the caregiver were examined by a hierarchical regression analysis: in the first block, professional sociodemographic variables were entered; in the second block, awareness of the risks of caregiving was added. Exploratory factor analysis (EFA) was used to identify the factor structures of responses. Internal consistency of scale responses was estimated using Cronbach’s alpha coefficient.

The linear interpolation method was used to assign values for the missing responses. Missing data was estimated as fewer than 4% of all responses (missing at random) [28]. The SPSS software (V. 21) was used for data processing and analyses. Statistical significance was set at *p* < 0.05.

## Results

A total of 295 primary physicians were offered to participate in the study, 201 of whom responded positively, representing a response rate of 68% (201/295). Hence, we interviewed 201 physicians for this study. The mean age of the physicians was 49 years, and most were female. Nearly half were born and graduated in Israel and most defined themselves as secular. Most of the physicians specialized in family medicine, were employed in HMO clinics, and gained an average experience of about 16 years of seniority in primary care practice. (Table 1).

**Table 1:** Socio demographic and professional characteristics of the primary physicians (*n* = 201)

Variable	Result
**Age (years)**	
Mean ± SD	48.5 ± 11.2
Range	28–77
**Number of children**	
Mean ± SD	2.4 ± 1.8
Range	0–12
**Gender [n (%)]**	
Males	93 (46.5)
Females	107 (53.5)
**Family status [n (%)]**	
Married/cohabits	93 (46.7)
Other	107 (53.5)
**Country of birth [n (%)]**	
Israel	97 (48.5)
Other	107 (53.5)
**Degree of religiousness [n (%)]**	
Secular	135 (67.2)
Traditional	28 (13.9)
Religious/ultra-religious	38 (18.9)
**Experience as a physician (years)**	
Mean ± SD	18.7 ± 12.2
Range	0.5–44
**Experience as a primary care physician (years)**	
Mean ± SD	16.2 ± 11.0
Range	0–44
**Number of weekly working hours**	
Mean ± SD	38.6 ± 15.9
Range	10–65
**Country of medical school graduation [n (%)]**	
Israel	96 (48.5)
Other	102 (51.5)
**Board certified [n (%)]**	
No	31 (15.8)
Yes	165 (84.2)
**Field of specialization [n (%)]**	
Family medicine	151 (85.3)
Other	26 (14.7)
**Place of work [n (%)]**	
Independent clinic	48 (23.9)
HMO clinic	138 (68.7)
Other	14 (7.0)

Relating to the physicians’ own family, about 39% had dependent elderly family members, and 34% had been primary caregivers for severely ill elderly family members with a mean duration of 44.3 ± 57.2 months of caregiving.

Exploratory factor analysis (EFA) with Varimax rotation performed on responses to each of the three domains of the physicians’ awareness to caregivers’ health and preventive treatment scale yielded only one factor. Internal consistency of the different domains, measured by Cronbach’s alpha, was found to be adequate: α = 0.79 for awareness, α = 0.65 for preventive treatment and α = 0.74 for monitoring (Table 2). Higher alpha values could not be achieved by deleting any items.

**Table 2:** Descriptive statistics of the three indices of the physicians’ awareness to caregivers’ health and preventive treatment scale and their internal reliability (n = 201)

Index measured^a^	No. of items	Range^a^	Mean (SD)	Median	α
Physicians’ awareness of caregiving risks ^b^	7	1–6	4.53(0.82)	4.71	79.
Physicians’ recommendations for preventive care ^b^	4	1–6	4.77(0.98)	5.00	65.
Monitoring of the primary caregiver ^c^	3	3–9	5.67(1.58)	6.00	74.

^a^ In all indices the scale direction is from lowest to highest

^b^ Calculated as the average score of the items

^c^ Calculated as the sum of the items

Most of the physicians identified that the caregiver of a severely ill or disabled family member was at high risk for morbidity or mortality. In addition, most of the physicians recommended treatment for the caregiver often (39.8%), or always (26.0%), to prevent a decline in their health condition. The utmost recommended treatments were regular physical activity followed by good sleep habits. The physicians also advocated that the caregiver seek professional help from a social worker or a psychologist approximately in one third of the cases. Most physicians (60.5%) stated that they invited caregivers for a follow-up visit on their own initiative. Moreover, regular clinic appointments for the caregiver were initiated by 56.6% of the physicians over the last six months.

Various associations were found between the three study indices and the physicians’ socio-demographic and professional characteristics. Female physicians were more likely to recommend preventive care than their male colleagues (*p* < 0.05). Israeli medical graduates were more aware of preventive care compared to those who studied abroad (*p* < 0.01), but abroad medical graduates were more likely to recommend preventive care compared to those who studied in Israel (*p* < 0.01). Board certified physicians were more liable to make recommendations for preventive care compared to non-board certified physicians (*p* < 0.01), and family physicians were more aware of caregiver health risks than non-family physicians (*p* < 0.001).

Finally, physicians who had the experience of being caregivers themselves were more likely to recommend preventive care than those who were not (*p* > 0.05). There were no statistically significant differences in physicians’ awareness, recommendations for preventive care, and in the follow-up treatment related to level of religiosity, family status, country of birth or place of work. It should also be noted that all associations between the physicians’ characteristics and the follow-up treatment were found not significant.

We conducted a multivariate linear regression analysis to determine the unique relative contribution of the study variables in explaining the physicians’ variability in awareness of the health risks of caregiving (Table 3). To the regression equation were added all variables found to be significant in the bivariate analyses. Since the variable “duration of caregiving” for a sick family member was significantly associated with risk awareness and only one third of the study physicians answered this item, it was added to the regression analysis as a dichotomous variable referring to whether the physician himself was a caregiver for a severely ill family member. The regression model was found to be significant (F[3172] = 6.65, *p* < 0.001). The following two variables, listed in ascending order, made a unique contribution to physicians’ awareness: Board certified family physicians and physicians who graduated medical schools in Israel were more aware of the risks of caregiving compared to the others. In total, these disparities explained a relatively low percentage (11%) of the variance of physicians’ awareness. Surprisingly, being a caregiver for a sick family member did not contribute significantly to the explanation of physicians’ awareness of the risks of caregiving.

**Table 3:** Results of multivariate linear regression analysis to determine variables that explain physicians’ awareness of the risks of caregiving (n = 201)

Variable	B	S.E	β	t
Being a primary caregiver^a^	.00	14.	.00	0.01
Country of medical school graduation ^b^	**34-	13.	−.20	**2.59-
Field of board certification^c^	**52.-	.17	−.22	**3.05-

**R**^**2**^ **= 0.11**

** *p* < 0.01

^a^ Being a primary caregiver – 1-Yes. 2-No

^b^ Country of medical school graduation – 1-Israel, 2-Other

^c^ field of board certification − 1-Family, 2-Other

In order to determine the unique relative contribution of the study variables to the explanation of physicians’ recommendations for preventive treatment, we conducted a hierarchical regression analysis (Table 4). In the first step, the socio-demographic and professional variables - gender, country of medical school graduation, board certified physician and being a primary caregiver- were entered simultaneously into the equation. Only board certified emerged significant; Board certified physicians recommended more preventive treatment than non-board-certified physicians. Country of medical school graduation approached the level of statistical significance (*p* = 0.08). The model explained 8.5% of the observed variance and was found significant (F[4, 185] = 4.27, *p* < 0.0010). In the second step the physicians’ awareness of the risks of caregiving was entered into the equation. This resulted in a statistically significant change in R^2^ (F[1185] = 17.6, *P* < 0.001), with an addition of 8% (ΔR2 = 8.0%). Together, the variables in the equation explained 16.5% of the observed variance (F[5, 184] = 7.24, *p* < 0.001). Three variables made a significant contribution for the explanation of physicians’ recommendations for preventive treatment: physician’s awareness of the risks of caregiving, country of medical school graduation and being a board certified physician (Table 4).

**Table 4:** Results of hierarchical multivariate linear regression analysis for explaining physicians’ recommendation for preventive treatment (n = 201)

Variable	B	S.E	β	t	R^**2**^	∆ R^**2**^
**First block**						
Board certified^a^	49.	20.	18.	**2.46		
Country of medical school graduation^b^	26.	15.	13.	1.71		
Gender^c^	22.	14.	11.	1.57		
Being a primary caregiver^d^	16.-	16.	07.-	1.00-	0.085***	
**Second block**						
Board certified ^a^	38.	19.	14.	*2.00		
Country of medical school graduation^b^	39.	15.	19.	**2.61		
Gender^c^	15.	14.	07.	1.07		
Being a primary caregiver^d^	17.-	15.	08.-	1.12-		
Awareness of the risks of caregiving	36.	09.	29.	4.19***	0.165***	0.08***

* *p* < 0.05, ** *p* < 0.01, ***p < 0.001

^a^ Board certified – 1-No, 2- Yes

^b^Country of medical school graduation – 1-Israel, 2-Other

^c^ Gender 1- Male, 2- Female

^d^ Being a primary caregiver 1-Yes 2. No

We did not conduct a regression analysis on physicians’ follow-up treatment because no independent variable was significantly associated with it in the bivariate analyses.

## Discussion

The main goals of the present study were to evaluate the extent to which PCPs are aware of family caregivers’ risks, and whether they take preventive measures to maintain caregivers’ health.

We found that, while a high percentage of physicians reported that they were aware of the caregivers’ higher risk for physical and mental impairments due to caregiving, they did not initiate interventions to address this vulnerable group. The reason for these inconsistent findings could stem from barriers such as lack of time, lack of knowledge, a different outlook, or other obstacles. Nonetheless, PCPs were aware of this issue, especially board-certified family physicians, so this could be a first stage in promoting appropriate treatment for caregivers in primary care medicine.

The professional literature focuses particular attention on caregivers for patients with dementia, since this large group of patients in the community places a heavy and prolonged burden on family members and on the healthcare system. For example, a Belgian study investigated the attitudes of PCPs to family caregivers for Alzheimer’s disease patients [24]. They found that physicians had a high level of skills and awareness of their role. The physicians expressed difficulty in reporting the diagnosis of dementia to patients and their family members and identified implementation barriers, especially lack of time and lack of appropriate remuneration. Despite their high level of awareness to the importance of treating caregivers, the same Belgian study found that caregivers were dissatisfied with the provision of home care [24].

Another study, conducted in Montreal, Canada, assessed the attitudes of family physicians to family members of elderly patients. They found that over 90% thought that their role was to address the requests and concerns of patients’ family members and felt that they fulfilled this role appropriately. However, 81% reported that this role caused work stress, deriving from the risk of diagnostic errors, conflict between the benefit of the patient and that of the family caregiver, or refusal on the part of the patient or family member to accept help from community organizations [29].

Our study evaluated whether PCPs reported providing recommendations to family caregivers in terms of preventive care. We found a low level of physician-initiated interventions to monitor the caregivers’ health. A recent review study assessed the effectiveness of intervention programs for family caregivers of patients with dementia. The authors concluded that there was no proof of the effectiveness of intervention programs in improving the physical health and social conditions of the primary caregivers [27].

Board certified family physicians and physicians who graduated medical schools in Israel contributed significantly to higher awareness level. This finding can be explained by the specialization curriculum in family medicine that puts an emphasis on the biopsychosocial approach which treats the patient and his family with a broad holistic attitude, integrating psychosocial factors related to health and not merely biomedical aspects of diseases. Moreover, the importance of informal caregiving is also taught as part of this curriculum and the subject is highly discussed in family medicine journals, textbooks and in professional conferences [30, 31]. As for physicians who graduated medical schools in Israel, a comprehensive study revealed that the curriculum of many Israeli medical schools includes a substantial part of social medicine education [32].

A surprising finding in our study was that physicians who were themselves caregivers for elderly or sick family members demonstrated a lower level of awareness to the needs of caregivers as the period of caregiving increased. A possible explanation for this finding might be the physician’s denial of the problem, or that, from the physician’s perspective, the role of the caregiver is considered as a “normal”, “natural” task that does not necessitate a special or specific attention. It is also possible that as the time of looking after a sick family member lengthened, the caregiver-physicians adapted and found ways to relieve their own burden.

Physician’s higher awareness of the risks of caregiving, country of medical school graduation (outside Israel) and being a board certified physician contributed significantly to physicians’ recommendations for preventive treatment. An interesting finding was that physicians who graduated outside of Israel made more preventive treatment recommendations than those who graduated in Israel. Similar to this finding, a recent study reported that physicians who graduated outside Israel refer their patients more to medical examinations compared with physicians who graduated in Israel [33]. This may stem from a sense of insecurity or from their medical education background. As for board certified physicians, as mentioned above, probably all curriculums of primary care specializations put an emphasis on treating patients and their family relatives in light of a broad biopsychosocial holistic approach. This may explain their higher recommendations for preventive treatment compared to non-board-certified physicians.

A secondary goal of this study was to develop a scale to measure physicians’ awareness to caregivers’ health and preventive treatment. Besides the face validity of the scale, the results of the statistical examination demonstrate that the items in each of the scale’s domains coalesce as one factor with satisfactory internal consistency. Future research should be conducted to demonstrate the validity of scale responses with larger and heterogenic samples.

### Study limitations

This was an observational study based on one-time interviews with a convenient sample of PCPs. Hence, causality cannot be inferred. It is possible that physicians who agreed to participate were more aware of the study topics, had a higher degree of interest or self-efficacy. Support for this assumption may be that many were older and more experienced physicians, as well as board-certified family physicians that had personal experiences as caregivers. Although the physicians were chosen from all geographic regions and HMOS, it is not clear whether the findings can be generalized to the entire population of physicians. Interviews might also reflect a social-professional desirability bias that could lead to answering in what they perceived as “was expected”. This could become particularly salient in questions on the extent of awareness and less on actual implementation of actions. Despite this potential tendency, we believe that the findings of the study are valid, and that, to a similar degree, this bias existed among all physicians.

## Conclusions

It is encouraging to notice the awareness to health risks and the willingness of primary care physicians to recommend treatment care to caregivers. This needs to be further nurtured and encouraged. Healthcare professionals/policy makers should be aware of the risks inherent in caregiving and should allocate support resources, such as the development of interventions at the community level. Special attention should address the most vulnerable groups such as female caregivers, elderly partners of patients, and caregivers who themselves have poor health. Intervention programs, such as skills training for family caregivers [34], are mainly aimed for the caregivers. We suggest, as Parmar and colleagues have developed [35], that such training should complement with courses for physicians and other primary care professionals to support the caregivers, enhance their wellbeing, enrich possible resources, and reduce the burden and costs for the healthcare services and for society.

As the population ages, especially in times of “viral lockdown”, as we experience globally in the last year, the number of family members fulfilling the role of caregivers is expected to increase. A recent research in the UK found higher rates of depression among family caregivers compared to non-caregivers, during the COVID 19 pandemic, with loneliness a significant contributor to depressive symptomatology [36]. Yet, most caregivers did not access any online or phone psychiatric support. This makes the front-line primary care physicians and staff even more significant as a support system in these difficult times.

## Supplementary Information

**Additional file 1: Supplementary material**.

## Data Availability

The datasets generated during and analyzed during the current study are not publicly available due to the sensitivity of the data but are available from the corresponding author on reasonable request.

## Abbreviations

WHO: World Health Organization
PCP: Primary Care Physicians
PCRS: Physician Caregiver Relationship Scales
