# Supplementary material for: Care for caregivers- a mission for primary care
Source: BMC Fam Pract. 2021 Nov 16;22:227. doi: 10.1186/s12875-021-01579-6 (PMC8593856; doi:10.1186/s12875-021-01579-6)
Supplement: Supplementary file 1 — Additional file 1: Supplementary material. [file 12875_2021_1579_MOESM1_ESM.docx]

**Care for Caregivers- a mission for primary care**

Aya Biderman MD^1,2^, Sara Carmel PhD^3,4^, Shimon Amar MD^1,2^,

Yaacov G. Bachner PhD^3,4^

**Supplementary information file**:

**Supplementary file 1**

***Table A.***

Items included in the index for physicians’ **awareness of the risks of caregiving** for primary caregivers

1. In your work as a physician do you inquire as to the identity of the primary caregiver in the family of a seriously ill or handicapped patient who is under your care?

2. Do you think that the primary caregivers of elderly patients are themselves at high risk for physical illness due to their caregiving?

3. Do you think that the primary caregivers of elderly patients are themselves at high risk for mental illness due to their caregiving?

4. Do you think that the primary caregivers of elderly patients who are elderly themselves are at high risk to die due to their caregiving?

5. Should family physicians relate to primary caregivers for seriously ill patients as a high-risk group for morbidity and mortality?

6. Is it important to invite primary caregivers on a regular basis for medical tests and follow-up?

7. Do you think that it would be beneficial for doctors of primary caregivers to arrange for a regular program of treatment and follow-up?

**Likert scale**: 1-6: No, Usually not, Occasionally, Sometimes, Often, Always

***Table B.***

Items included in the variable **recommendations for preventive treatment** for primary caregivers

1. Do you recommend to the primary caregiver to adopt a lifestyle that will prevent deterioration in their health condition?

2. Do you recommend physical exercise?

3. Do you recommend appropriate sleep habits?

4. Do you notify the primary caregiver that they can get help from a social worker/psychologist?

**Likert scale**: 1-6: No, Usually not, Occasionally, Sometimes, Often, Always

***Table C.***

Items included in the variable **treatment follow-up** for primary caregivers.

1. In your daily routine, do you invite primary caregivers for clinic visits even if they do not request it?

2. Over the past six months did you inquire, at your own initiative, about the physical or mental health of any primary caregiver?

3. Over the past six months did you invite, at your own initiative, a primary caregiver to the clinic out of concern for their health?

**Likert scale**: 1-3: Never, Several times, Often
